# Does proteopathic tau propagate trans-synaptically in the brain?

**DOI:** 10.1186/s13024-022-00527-x

**Published:** 2022-03-16

**Authors:** Wen Hu, Fei Liu, Cheng-Xin Gong, Khalid Iqbal

**Affiliations:** Department of Neurochemistry, Inge Grundke-Iqbal Research Floor, New York State Institute for Basic Research in Developmental Disabilities, Staten Island, New York, USA

Neurofibrillary pathology comprising hyperphosphorylated tau is one of the two hallmarks that characterize Alzheimer’s disease (AD) histopathologically, and it is clinically correlated with, and predicts the severity of, cognitive deficits in AD. The spread of tau pathology within AD brain follows a stereotypical pattern, from the trans-entorhinal region to the limbic system and eventually to the primary cortical areas. This hierarchical pattern suggests that tau pathology is transmitted from one area of the brain to other regions via anatomical connection [1].

The transmission of tau pathology is attributed to the propagation of seeding-competent/proteopathic tau from neuron to neuron and the resultant prion-like templated aggregation in the recipient cell. Several hypotheses explain how proteopathic tau spreads from one brain region to another. The most popular one is the trans-synaptic hypothesis, proposing that proteopathic tau propagates via the synapse in an anterograde fashion [2–4]. However, a comprehensive histopathological study of postmortem human brains showed that the progression of dendritic tau lesions in the temporal allocortex appeared to follow a direction opposite to currently known unidirectional hippocampal connectivity despite that rare or unknown projections might be involved [5]. Does proteopathic tau propagate trans-synaptically in the brain?

The trans-synaptic hypothesis initially proposed was based on studies using bigenic neuropsin-tTA-tau mice [2, 3]. The bigenic model was generated by crossing two existing mouse lines, the neuropsin-tTA transactivator line (the tTA-EC line) and the Tg(tetO-tau_P301L_)4510 responder line. In this bigenic model, the aggregation-prone, P301L-mutated human tau was expressed predominantly in the superficial layers of the medial entorhinal cortex (EC) and pre−/para-subiculum. It appeared to take several months for somatodendritic tau pathology to progress from the EC to the granule cell layer of dentate gyrus (GrDG), a hippocampal subfield that is known to unidirectionally receive axonal inputs from the EC.

Intriguingly, the removal of endogenous mouse tau in the bigenic neuropsin-tTA-tau model did not appear to affect EC-GrDG tau “propagation” [6]. This finding suggests that late-onset tau pathology in the GrDG was a consequence of accumulation of “propagated” tau from the EC if, indeed, propagation did occur. It also suggests that no prion-like mechanism was involved. Another possibility could be that tau propagation did not occur in this model. Indeed, the responder line itself exhibited a certain level of “leaky” expression of human tau in the dentate gyrus in the absence of tTA transgene [7]. In addition, a detailed brain-wide survey of the distribution of the tTA transgene in the tTA-EC activator line revealed that tTA expression was not inherently restricted to the EC but instead distributed in a broad fashion; a subset of DG granule cells also exhibited appreciable levels of tTA [8]. Therefore, the delayed development of somatodendritic tau pathology in the GrDG in neuropsin-tTA-tau mice could result from low expression of human tau independently of tau propagation, because the expression level of aggregation-prone tau predicts the rate of development of tau pathology. Thus, data from the neuropsin-tTA-tau mouse model are not sufficient to substantiate a definite conclusion of trans-synaptic tau propagation.

A model that is more disease-relevant and persuasive in testing trans-synaptic tau propagation is the in vivo tau inoculation model, in which tau pathology is induced and characterized in the mouse brain by injection of proteopathic tau. Although neuronal connectivity-based propagation was consistently observed [9–13] and inter-neuronal tau propagation was thought to occur in some inoculation studies, detailed analyses of the representative data do not appear to support the trans-synaptic hypothesis [9–11, 14]. First, up to 11 months after tau inoculation in the hippocampus, somatodendritic tau pathology was induced locally and in axonally connected upstream (afferent) regions, including the superficial layers of the EC [9, 15] and the medial septal nucleus [11]; only axonal/neuritic—no somatodendritic—tau accumulation was seen in distant regions that are exclusively or predominantly downstream (efferent) to the hippocampus, including the deep layers of the EC [9], the lateral septal nucleus [9, 11] and the contralateral hippocampal CA1 subfield [11] (Fig. 1A). Second, detailed surveys of tau pathology in the whole brain after tau inoculation in non-transgenic mice and computational modeling showed that pathological tau mostly underwent retrograde propagation to distant regions according to known brain connectivity [10, 11, 15]. Third, tau inoculation in the hippocampus consistently induced tau pathology in the locus coeruleus within 2 weeks in young adult PS19 (tau_P301S_) mice; in contrast, tau inoculation in the locus coeruleus failed to induce tau pathology in the hippocampus, even after incubation for 6 months under experimental conditions that were otherwise identical [14] (Fig. 1B). Locus coeruleus is a pontine nucleus that projects axons to, but does not receive axonal inputs from, the hippocampus. Therefore, there is no evidence of secondary seeding due to trans-synaptic propagation of tau from the hippocampus to EC deep layers or from the locus coeruleus to the hippocampus. Taken together, the in vivo tau inoculation studies suggest axonal uptake of injected tau and primary seeding in distant brain regions, but they do not support trans-synaptic tau propagation (Fig. 1C).

**Fig. 1:**
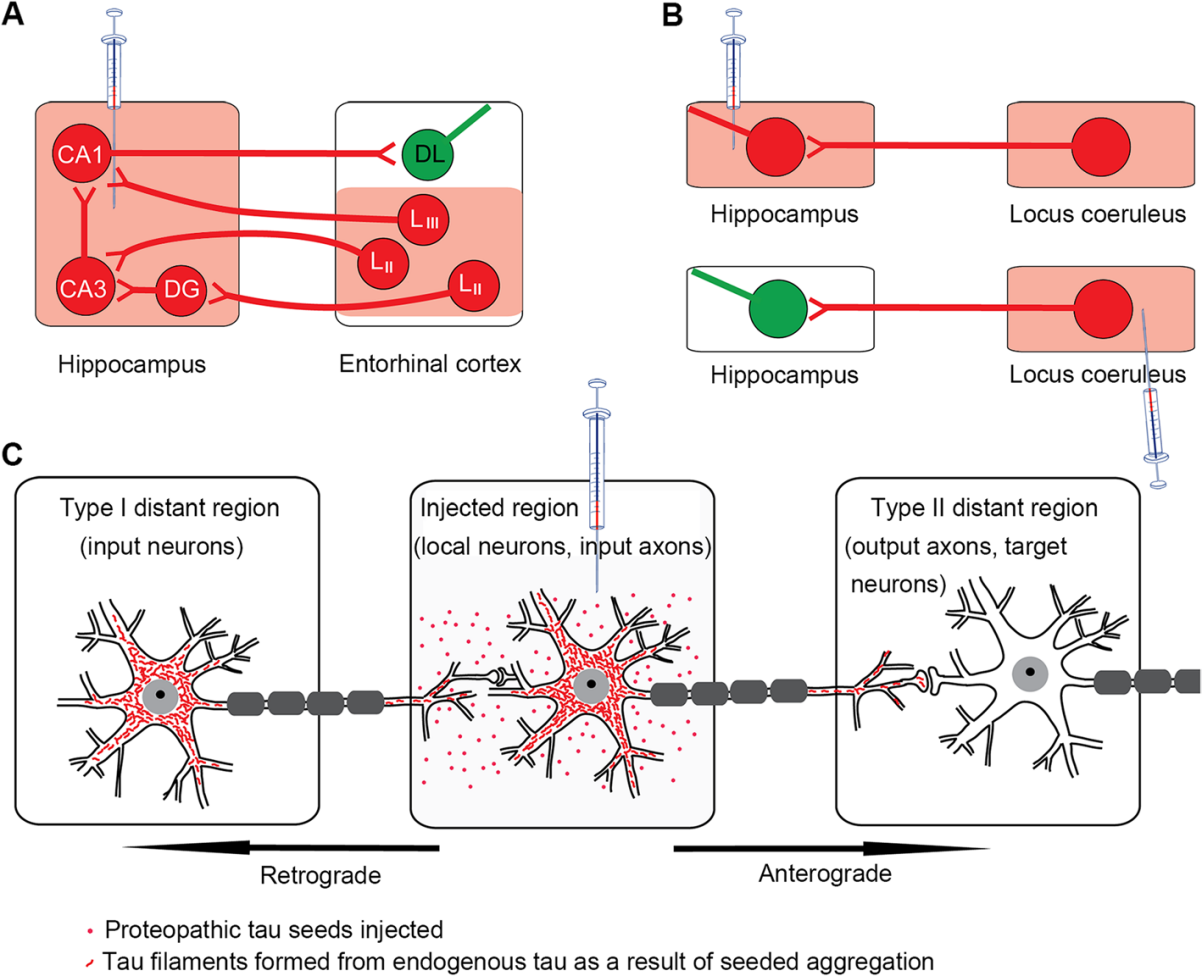
Injection of proteopathic tau seeds in the mouse brain induces somatodendritic tau pathology locally and in distant regions that project axons to the injected area. **A**, **B** Schematic diagrams showing neurons with their projection in the known neural circuit and the regions with induced tau pathology after tau inoculation. Projection axons and their terminals are shown as bold lines and bifurcations, respectively. Neurons exhibiting somatodendritic tau pathology and axonal tau accumulation are shown in red (the brain region/subregion in pink), and those free from somatodendritic tau burden in green. After injection of tau seeds into the hippocampus, layers II (L_II_) and III (L_III_) of the entorhinal cortex (EC) showed somatodendritic tau inclusions, whereas the EC deep layers (DL) only show axonal tau accumulation (**A**); the distinct patterns clearly demarcated the superficial layers from deep layers of the EC [9]. Tau pathology was induced in the locus coeruleus after tau inoculation in the hippocampus, but not vice versa (**B**) [14]. **C** Schematic diagram showing proposed model of induced tau pathology in the mouse brain after tau inoculation. Somatodendritic tau pathology is seen mostly in the injection area and type I distant brain regions in which neurons project axons to the injected region. Note that this category includes distant regions that are bidirectionally connected to the injected area. However, the type II distant region, where neurons receive axonal inputs from but do not project axons to the injected area, exhibits only axonal/neuritic tau pathology without somatodendritic tau burden

The synapse is an inter-neuronal contact area that is highly specialized for the flow, and its modulation, of neural signals between neurons. Other than neurotransmitters and neuromodulators, only a few neurophilic viruses and toxins are known to undergo trans-synaptic transmission. As a microtubule-associated protein that functions primarily to stimulate the assembly of, and help stabilize, microtubules, tau is thought to undergo axonal sorting and to be mainly localized in the axonal compartment of mature neurons under physiological conditions. Tau is also thought to be mis-localized and aggregated in the somatodendritic compartment under pathological conditions. Although tau is found at the pre- and post-synaptic compartments, it remains uncertain whether and how seeding-competent tau species, if generated somatodendritically, are transported anterogradely in the axon and released to the synaptic cleft.

Although the absence of convincing evidence does not exclude the possibility of trans-synaptic tau propagation, based on the experimental data accumulated so far, whether proteopathic tau propagates trans-synaptically in vivo remains a question, and a definite conclusion remains to be substantiated in future studies. Whereas tau gene delivery supplemented with 2A self-cleaving peptides has emerged as an attractive tool in differentiating donor and recipient neurons in the context of inter-neuronal tau transfer [6], the specificity of the technique remains to be validated. An ideal model to test trans-synaptic tau propagation would be intracerebral tau inoculation that targets a well-established unidirectional circuit in rodents and is free from confounding by endogenous tau load. The key determinant for a successful inoculation model is the precision injection of a small volume (10-100 nl, for instance) of proteopathic tau that is truly restricted to the target area, as was done in the early tract-tracing studies. Pressure injection of a large volume in the brain parenchyma can be problematic because of off-targeting of neurons that are irrelevant to the circuit of interest. In tau inoculation, simultaneous tracing with suitable neuronal tracers that exhibit labeling stability and minimal neural toxicity can be informative for better interpretation of data.

It takes years for tau pathology to progress to a higher Braak stage in the human brain. If trans-synaptic tau propagation does occur in the tau inoculation mouse model, it should involve the generation of secondary proteopathic tau in the neurons that have internalized the injected, primary seeds, and involve the extracellular release of the secondary seeds at the synapse and in turn the uptake of these seeds by synaptically connected neurons. In this regard, the incubation period should be substantially longer than primary seeding. Although transgenic mice overexpressing aggregation-prone mutated tau may enable rapid tau seeding, the transgene expression can be uneven in the brain, and the varying levels of expression should be carefully considered in interpreting inoculation studies [12, 14].

Inspired by the Braak staging of tau pathology, the conceptual evolution from intraneuronal accumulation of tau filaments to a more dynamic process that involves inter-neuronal trafficking of proteopathic tau via the extracellular space has pinpointed tau as a promising therapeutic target. Mechanistic insights into how AD tau pathology progresses would help guide the development of therapeutic strategies. Future studies are needed to ascertain whether proteopathic tau propagates trans-synaptically in a commonly thought anterograde, or possibly retrograde, fashion in the brain, and to unravel the mechanisms underlying the stereotypical pattern of tau pathology progression.

## Data Availability

Not applicable.

## Abbreviations

AD: Alzheimer’s disease
EC: Entorhinal cortex
GrDG: Granule cell layer of dentate gyrus
tTA: Tetracycline transactivator
